# Reactive Flash Sintering of High-Entropy Oxide (Mg, Co, Ni, Cu, Zn)_1−x_Li_x_O at Room Temperature

**DOI:** 10.3390/ma15113836

**Published:** 2022-05-27

**Authors:** Nianping Yan, Yuchen Zhu, Muliang Cai, Bojiang Li, Bichuan Xu, Yueji Li, Xilin Wang, Zhidong Jia

**Affiliations:** 1 State Grade Jiangxi Electric Power Research Institute, Nanchang 330096, China; ynphust@163.com (N.Y.); caiml2021@126.com (M.C.); 565052374@163.com (B.L.); xubichuan123@163.com (B.X.); 2 Engineering Laboratory of Power Equipment Reliability in Complicated Coastal Environments, Tsinghua Shenzhen International Graduate School, Tsinghua University, Shenzhen 518055, China; zhuyc15@mails.tsinghua.edu.cn (Y.Z.); liyueji1999@126.com (Y.L.); jiazd@sz.tsinghua.edu.cn (Z.J.)

**Keywords:** flash sintering, room temperature, high entropy ceramics, alternating current field (AC)

## Abstract

(Mg, Co, Ni, Cu, Zn)_1−x_Li_x_O is a type of high-entropy oxide that has high ionic conductivity at room temperature and is used as a solid electrolyte. (Mg, Co, Ni, Cu, Zn)_1−x_Li_x_O was successfully synthesized from precursor powder by applying reactive flash sintering for less than 4 min at room temperature (25 °C). AC and DC electric fields were independently applied to sinter ceramic samples; consequently, AC and DC electric field application resulted in relative densities that exceeded 90% and 80%, respectively. X-ray diffraction spectra of samples revealed the existence of a clear halite structure with an insignificant impurity phase, proving that (Mg, Co, Ni, Cu, Zn)_1−x_Li_x_O crystals were successfully produced.

## 1. Introduction

Flash sintering is a novel sintering method that was initially reported in 2011 by Cologna; it has the following advantage over conventional sintering methods: the green bodies of ceramics can be densified in less than 1 min at furnace temperatures that are several hundred degrees lower [1]. The shorter sintering time and lower furnace temperatures make flash sintering more energy-efficient than conventional sintering [2]. As a relatively new ceramic processing method, flash sintering has garnered considerable attention and has been applied to various types of materials [2,3,4,5,6,7].

In flash-sintering studies, researchers have adopted several methods to reduce the onset furnace temperature. For example, Zhang and Luo decreased the onset temperature of zinc oxide to 120 °C in a reduced atmosphere [8]. Down and Sglavo successfully flash-sintered 8YSZ at a furnace temperature of 390 °C by increasing the DC electric field to 2250 V/cm [9]. Wu et al. prepared ZnO varistor ceramics using the flash-sintering method at an air pressure of 21 kPa for 60 s at 25 °C [10]. In recent studies, our group demonstrated that dog-bone-shaped zinc oxide can be sintered at room temperature under the condition of an AC electric field of up to 3.13 kV/cm [11,12]. In the future, more materials are expected to be sintered at lower furnace temperatures.

The combination of reactive sintering and flash sintering resulted in development of a new technique called reactive flash sintering (RFS), wherein both phase transformation and ceramic sintering occur during the flash process [13,14,15,16]. Bola Yoon demonstrated that a mixture of Mg and α-Al_2_O_3_ powder can be transformed into MgAl_2_O_4_ ceramic material through the process of reactive flash sintering [13]. Furthermore, Murray et al. successfully fabricated a densified Mn_3_O_4_ ceramic from a Mn_2_O_3_ precursor powder by performing a one-step flash-sintering process [14].

High-entropy ceramics consist of at least five oxides and uniform crystal structures. Multiple compositions yield high-entropy ceramics with a variety of unique properties [17,18,19,20]. For example, (Mg, Co, Ni, Cu, Zn)_1−x_Li_x_O (0 < x < 1) is a high-entropy ceramic with high ionic conductivity at room temperature [21], making it a promising candidate for application as a solid electrolyte. To produce (Mg, Co, Ni, Cu, Zn)_1−x_Li_x_O via conventional sintering, the precursor powder needs to be heated at 1000 °C for approximately 12 h [17]. In consideration of this, a technique such as reactive flash sintering, which can ultimately reduce the required sintering temperature and time, would be beneficial.

Mao et al. successfully synthesized nearly fully dense high-entropy ceramics at 1200 °C in a short time [22]. Li et al. successfully synthesized high-enthalpy and high-entropy Ca_0.2_Co_0.2_Ni_0.2_Cu_0.2_Zn_0.2_O oxide ceramics at room temperature [23]. Cheng et al. successfully synthesized (Cu, Zn, Mg, Co, Ni)O by employing a reactive flash sintering technique [24]. In this study, we successfully synthesized (Mg, Co, Ni, Cu, Zn)_1−x_Li_x_O from a precursor powder by applying reactive flash sintering for less than 4 min at room temperature. After investigating the effects of AC and DC electric field application, the sintered ceramic samples had relative densities of more than 90% and 80%, respectively. Additionally, the X-ray diffraction (XRD) spectra clearly revealed a halite structure with minimal impurities, proving that (Mg, Co, Ni, Cu, Zn)_1−x_Li_x_O crystals were successfully produced.

## 2. Experimental Methods

Oxides and carbonates, i.e., MgO, Co_3_O_4_, Ni_2_O_3_, CuO, ZnO, and Li_2_CO_3_, were mixed in different stoichiometric amounts (for Li, x was set to 0.1 or 0.3) by applying planetary ball milling at 250 rpm for 60 min. Ethanol (99%) was also added as a dispersant to facilitate uniform mixing. Table 1 shows the detailed information on the powders used and the quantitative oxide composition of the produced mixture. Hereafter, 0.1Li or 0.3Li is used to represent the x = 0.1 or x = 0.3 stoichiometric samples, respectively. After drying the mixture at 120 °C for 4 h, a 10 wt.% polyvinyl butyral (PVB) solution (i.e., 10 wt.% PVB in DI water) was blended into the precursor powders. Samples of the mixture were then uniaxially pressed into dog-bone-shaped specimens under a pressure of 800 MPa. Green samples were heated at 360 °C for 4 h to remove the binder (with no obvious shrinkage). The results of geometric measurements revealed that the relative densities were approximately 57% and 65% for the 0.1Li and 0.3Li green bodies, respectively. The length of each of the dog-bone samples was 14.4 mm, and the cross-sectional area was 5.58 mm^2^. After analyzing their geometry, both ends of all specimens were painted with GW02 silver paste and heated at 650 °C for 10 min to make silver electrodes.

During the reactive flash-sintering process, green samples were placed on an alumina plate to prevent discharge from the samples to the ground. Pt wires were twisted around the silver electrodes on the samples and connected to an AC or DC power supply. A digital camera was used to monitor the flash-sintering process. The maximum output voltage of the 50 Hz AC power supply (YDTW-100/50, Xinyuan Electric Co., Ltd.; Yangzhou, China) used in the experiment was 50 kV; additionally, the initial voltage was constant at 900 V, and its rated output current was 2 A. (Note that all values are root-mean-square (RMS) values.) The highest output voltage and rated current of the DC power supply (FTG100-1500, Faithtech Co., Ltd.; Shenzhen, China) were 1500 V and 7 A, respectively. Note that the DC and AC power supplies were each allocated their own output voltage and current monitoring equipment. It is also important to note that the dog-bone sample tended to fracture if the current was increased too quickly. Thus, in our flash-sintering experiment, the current increasing speed was limited.

In the AC flash sintering experiments, the amount of voltage initially applied to the dog-bone samples was 900 V, corresponding to an electric field of 625 V/cm. A few seconds after switching on the power, flash sintering was observed, as the voltage decreased to less than 72 V (50 V/cm), and the current density increased from 0 to approximately 2 A/cm^2^. Then, the power output of the test power supply was increased to increase the output current density. Approximately 2 min thereafter, the current across the dog-bone samples increased to approximately 0.8 A (14.34 A/cm^2^). After maintaining the 0.8 A output current for 1 min, the AC test power supply was disengaged. (All of the voltage and current values mentioned above are the RMS values.)

An initial voltage of 400 V (278 V/cm) was applied in the DC flash-sintering experiment. The initial current limit was 0.2 A (current density 3.46 A/cm^2^) for the 0.1Li samples, and 0.1 A (current density 1.73 A/cm^2^) for the 0.3Li samples. The initial conditions were maintained for 20 s, during which time, flash sintering occurred, and the DC power supply was switched from constant voltage mode to constant current mode. Then, the voltage remained unchanged as the current limit was increased by 0.2 A (3.46 A/cm^2^) or 0.1 A (1.73 A/cm^2^) in intervals of 10 s until the preset maximum current of 0.8 A (14.34 A/cm^2^) or 1 A (17.93 A/cm^2^) was reached for the 0.1Li samples or 0.3Li samples, respectively. The maximum current was maintained for 60 s before the DC power supply was disengaged.

Voltage and current changes that occurred during the flash-sintering process were recorded in real time; additionally, a thermal infrared imager (SC660, Teledyne FLIR Co., Ltd.; Wilsonville, OR, USA) was used to monitor the temperature of the samples during the flash-sintering process. The relative densities of the sintered samples were measured by applying the Archimedean method (the reference theoretical densities of the 0.1Li and 0.3Li samples were 5.221 and 4.997 g/cm^3^, respectively); their structures were analyzed by applying XRD (D8 Advance, Bruker Corp.; Billerica, MA, USA) and XPS (PHI 5000 Versa Probe II, ULVAC-PHI Inc.; Tokyo, Japan). The fractured surfaces of these specimens were examined by using a scanning electron microscope (SU8010, Hitachi Co., Ltd.; Tokyo, Japan), and the ionic conductivity of each sample was calculated based on the electrochemical impedance spectral results, which were obtained by using a broadband dielectric spectrometer (Concept40, Novocontrol Technologies GmbH & Co.KG; Montabaur, Germany).

## 3. Results and Discussion

**Figure 1:**
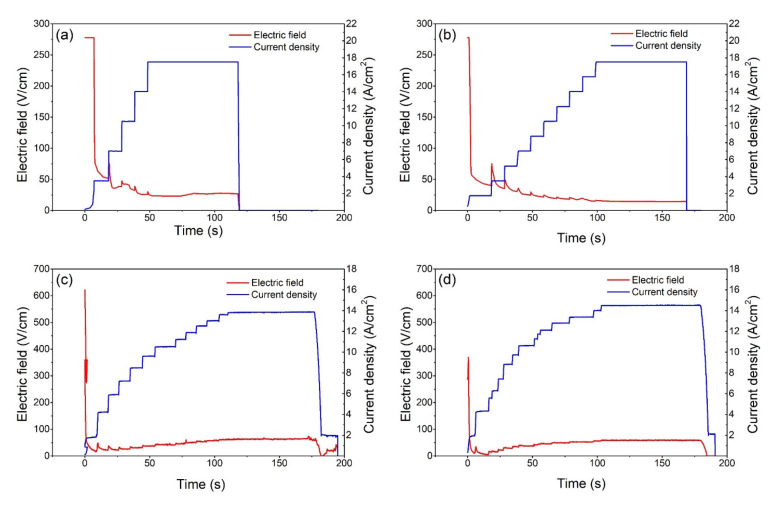
Electric field and current density change during (**a**) 0.1Li DC, (**b**) 0.3Li DC, (**c**) 0.1Li AC, and (**d**) 0.3Li AC flash sintering.

Figure 1 illustrates the changes in the electric field and current density over time as the 0.1Li and 0.3Li samples underwent DC and AC flash sintering. The curves clearly show that the magnitude of the electric field (or voltage) abruptly decreased to a very low value after the flash occurred, regardless of the current limits; this observation is consistent with those observed in previous flash-sintering studies [11]. In all cases, the current density was subject to small fluctuations in response to an increase in the current; this is because the conductivity of the samples did not change as fast as the electric parameters. As the current limit (DC) or power output (AC) was gradually increased, the magnitude of the electric field tended to further decrease during the DC flash-sintering process; alternatively, during the AC experiments, the magnitude of the electric field tended to slightly increase. Additionally, the final electric field strength of the 0.1Li specimens tended to be slightly higher than that of the 0.3Li specimens, proving that the conductivity of the 0.1Li samples was lower in the flash state and that the 0.1Li samples retained more electric power.

**Figure 2:**
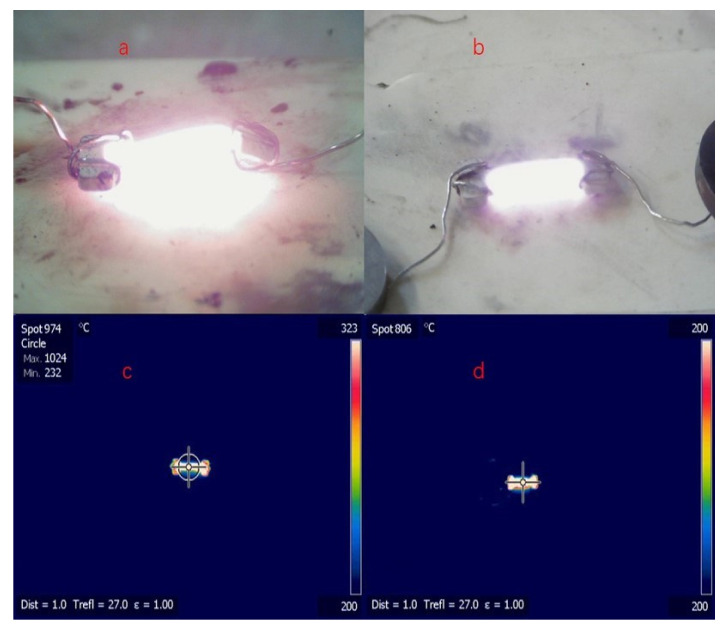
Digital camera: (**a**) 0.3Li, AC, 0.8-A maximum current; (**b**) 0.3Li, DC, 1-A maximum current; thermal infrared imager: (**c**) 0.3Li, AC, 0.8-A maximum current; (**d**) 0.3Li, DC, 1-A maximum current.

A digital camera was used to monitor the flash-sintering process, and the temperature of each ceramic sample was monitored by using thermal infrared spectroscopy, whereas the theoretical temperature was calculated by using a blackbody radiation model [25]; the results are presented in Figure 2 and Table 2. The measured temperatures tended to be lower than the calculated temperatures, which suggests that the power dissipation was larger than the blackbody radiation loss. Thus, the heat conduction that occurs during flash sintering may be non-negligible and should therefore be carefully considered when estimating the steady-state temperature of ceramic samples. Furthermore, because there was less electric power in the steady state, the sintering temperature of the 0.3Li samples tended to be lower than that of the 0.1Li specimens, and the DC mode also yielded comparatively lower-temperature specimens, even under the condition of a larger current.

Table 2 also summarizes the relative density results for the (Mg, Co, Ni, Cu, Zn)_1−x_Li_x_O (x = 0.1 or 0.3) specimens sintered under different conditions. As can be seen, the relative density of the AC-sintered samples exceeded 90%, whereas that of the samples sintered via DC voltage application exceeded 80%, but did not reach 90%, even though the steady-state current was larger. A sigher sample temperature during AC flash sintering promotes the densification of samples. The AC waveform may have also facilitated densification during the AC flash-sintering experiments due to the higher voltage instantaneous value. Moreover, constant changes in the direction of the electric field may speed up the chemical reaction and makes it more complete. All relative densities of the specimens with x = 0.3 were lower than those of the specimens with x = 0.1; this is partially attributed to the higher temperatures reached by the 0.1Li specimens, as mentioned above. Less Li_2_CO_3_ decomposition results in less heat absorption, which is one of the reasons for the higher temperature. Moreover, the higher fragility of the 0.3Li samples required smaller incremental increases in the current limits during the DC flash-sintering process than the 0.1Li samples; this may also have contributed to them having lower relative densities.

The steady-state temperatures observed during the flash-sintering process were close to conventional sintering temperature (i.e., 1000 °C or 1273.15 K); however, the relative densities of the ceramic specimens increased by more than 20% in 3 min; this level of increase typically takes several hours under the conditions of conventional sintering. Thus, high temperatures were not the only reason for the faster densification and solid-state reaction in our experiment. The external electric field, current, and rapid temperature increases also facilitated the entire process [2]. However, the specific flash-sintering principle of (Mg, Co, Ni, Cu, Zn)_1−x_Li_x_O remains unknown, and needs to be further explored.

**Figure 3:**
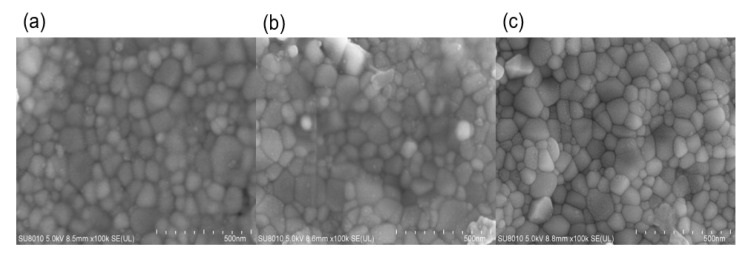
SEM images of (**a**) positive pole; (**b**) negative pole; (**c**) middle.

Figure 3 shows the SEM images of the 0.1Li specimens that were flash-sintered under the conditions of DC and a 1.0 A maximum current. The sample was relatively dense and the density at the positive pole was higher than that at the negative pole. There was a higher potential difference between the positive electrode and air, so oxygen was more likely to participate in the reaction at the positive electrode, which may be the reason for the higher density at the positive electrode.

**Figure 4:**
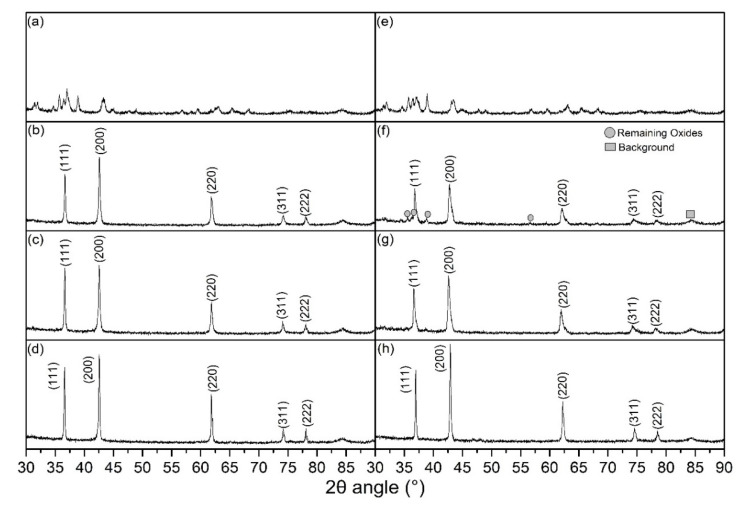
XRD spectra of ceramic samples sintered under different conditions: (**a**) 0.1Li green bodies; (**b**) 0.1Li, DC, 0.8 A; (**c**) 0.1Li, DC, 1 A; (**d**) 0.1Li, AC, 0.8 A; (**e**) 0.3Li green bodies; (**f**) 0.3Li, DC, 0.8 A; (**g**) 0.3Li, DC, 1 A; and (**h**) 0.3Li, AC, 0.8 A.

Figure 4 shows the XRD patterns of the 0.1Li and 0.3Li specimens that were flash-sintered under different conditions. It is clear that a nearly pure phase [17,21] (Mg, Co, Ni, Cu, Zn)_1−x_Li_x_O with a halite structure was successfully synthesized via the DC and AC reactive flash-sintering methods. Only a small portion of the oxides remained in the 0.3Li specimens that were sintered under the conditions of DC and a 0.8 A maximum current. Thus, the XRD spectra prove that reactive flash sintering is an effective way to produce single-phase (Mg, Co, Ni, Cu, Zn)_1−x_Li_x_O ceramics. The ratio between the first and second peak values for all specimens was larger than 0.67, which is the theoretical value of an ideal halite structure; this indicates that there was Jahn–Teller deformation in these samples.

**Figure 5:**
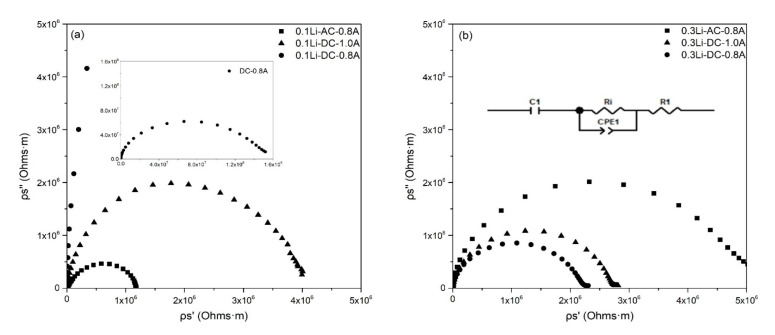
Room temperature impedance spectra of different samples. (**a**) 0.1Li sample impedance spectra; (**b**) 0.3Li sample impedance spectra and equivalent circuit.

The room-temperature impedance spectra of the ceramic samples sintered under different conditions are presented in Figure 5. The electronic conductivity of (Mg, Co, Ni, Cu, Zn)_1−x_Li_x_O is lower than 5 × 10^−9^ S/cm and can thus be ignored [21]. The equivalent circuit used to estimate the ionic conductivity of the samples is shown in the inset in Figure 5b. Ri represents the ionic resistance of (Mg, Co, Ni, Cu, Zn)_1−x_Li_x_O, CPE1 originates from charge gradients, R1 is the contact resistance between electrodes and sintered (Mg, Co, Ni, Cu, Zn)_1−x_Li_x_O, and C1 represents the blocking of ions on the surfaces between sintered ceramics and electrodes. According to the fitting result (Ri) of the equivalent circuit, the conductivity of sintered (Mg, Co, Ni, Cu, Zn)_1−x_Li_x_O was calculated by Formula (1):

(1) $$\sigma_{i}=\frac{d}{R_{i}S}$$

where S and d are the area and thickness of sintered samples, respectively. As listed in Table 1, the room-temperature ionic conductivities of flash-sintered samples were all lower than 10^−8^ S/cm, indicating that they were ionic insulators. The ion conductivity of flash-sintered (Mg, Co, Ni, Cu, Zn)_1−x_Li_x_O in our study is also much lower than that of samples in other research. There are two possible reasons for this unexpected result: (1) the remaining Co^3+^ compensated for one valence Li^+^ ion or (2) the oxygen at the positive pole eliminated the oxygen vacancies introduced by Li^+^.

**Figure 6:**
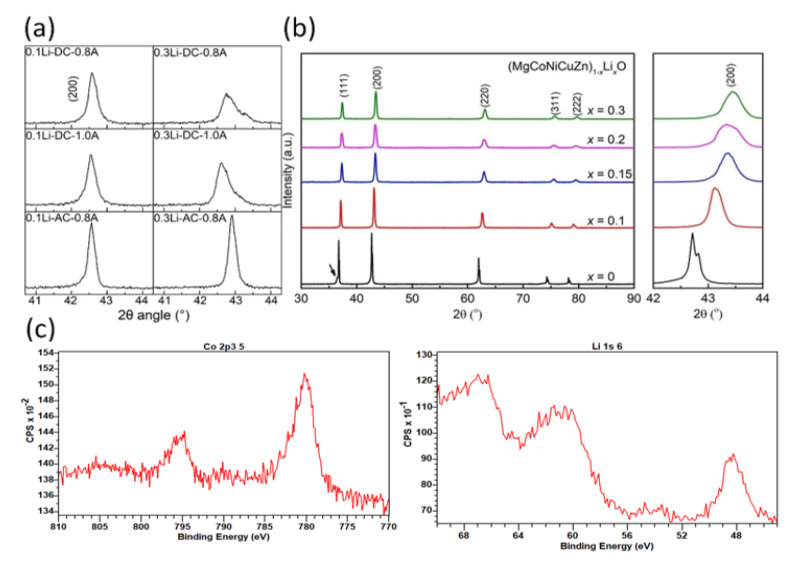
(**a**) The (200) peaks of samples sintered under different conditions in this study; (**b**) magnified view of the (200) peak showing the high-angle shift upon Li incorporation, flash-sintered under 735 mA/mm^2^ for 60 s, reproduced with the permission of ref. [26], copyright @2022, *Journal of American Ceramics;* (**c**) X-ray photoelectron spectroscopy (XPS) spectra of 0.1Li-DC-1.0A sintered samples.

To explain the low ionic conductivity, more tests are conducted. According to other studies, doping Li into (Mg, Co, Ni, Cu, Zn)O will lead to shrinkage, making the (200) peak shift to a higher angle, as shown in Figure 6b [26]. A larger content of Li can induce larger displacement of the (200) peak position. The (200) peak positions in our experiment are shown in Figure 6a. It is obvious that in our study, the peak position displacement for the same content of Li was much smaller compared with that in other research. The (200) peak positions exceeded 43° when Li reached 10 mol.%, as shown in Figure 6b, while all the (200) peak positions were less than 43° in our research, even if the Li content was more than 30 mol.%. So, the lattice shrinkage of our sintered (Mg, Co, Ni, Cu, Zn)_1−x_Li_x_O is much less than that reported in other research. Figure 6c demonstrates the XPS spectra of the flash-sintered samples. The characteristic satellite peak of Co^3+^ at 789 eV was not observed in any sintered ceramics, proving that all of Co is two valences. Thus, there was no Co^3+^ in our sintered samples compensating for Li^+^. Meanwhile, XPS analysis of the samples confirmed the existence of Li^+^ and the oxygen ratio reached 41.1%, indicating that oxygen participated in the reaction. Therefore, the low ionic conductivity of our sintered (Mg, Co, Ni, Cu, Zn)_1−x_Li_x_O is mainly due to the oxygen at the positive pole eliminating the oxygen vacancies introduced. Most of Li is located in the halite structure of sintered samples.

## 4. Conclusions

This study successfully synthesized halite-phase high-entropy ceramics (Mg, Co, Ni, Cu, Zn)_1−x_Li_x_O by applying reactive flash sintering at room temperature. The relative densities of the resulting ceramics exceeded 80% and 90% under the respective conditions of DC flash at 1 A and AC flash at 0.8 A (RMS), indicating that AC may be a better choice when flash sintering this type of material. Additionally, the samples with x = 0.1 were more easily densified than those with x = 0.3; this was attributed to the lower conductivity that was observed during flash sintering. Furthermore, the ionic conductivity of flash-sintered (Mg, Co, Ni, Cu, Zn)_1−x_Li_x_O was found to be extremely low; this may be attributable to the participation of O_2_, or the compensatory relationship between Li^+^ and the remaining Co^3+^ and Ni^3+^.

## Figures and Tables

**Table 1:** Detailed information on the powders. (mol.% on the oxide/carbonate).

Powers	Purity (%)	Particle Size (nm)	Producer	0.1Li Moore Content (%)	0.3Li Moore Content (%)
MgO	99.9	50	Shanghai Macklin Biochemical Co., Ltd., Shanghai, China.	18	14
Co_3_O_4_	99.5	30	Shanghai Macklin Biochemical Co., Ltd., Shanghai, China.	6	4.67
Ni_2_O_3_	99.0		Shanghai Macklin Biochemical Co., Ltd., Shanghai, China.	9	7
CuO	99.5	40	Shanghai Macklin Biochemical Co., Ltd., Shanghai, China.	18	14
ZnO	99.5	15	Nanjing Emperor Nano Material Co., Ltd., Nanjing, China.	18	14
Li_2_CO_3_	99.0		Shanghai Maikun Chemical Co., Ltd., Shanghai, China.	10	30

**Table 2:** Important parameters for specimens sintered under different conditions (Current values in “Experimental Conditions” represent the steady-state RMS currents during flash sintering.).

Experimental Condition	Relative Density (%)	Theoretical Steady-State Temperature (K)	Measured Steady-State Temperature (K)	Ionic Conductivity (S/cm)
0.1Li Green	57.46%			
0.1Li-DC-0.8A	82.88%			7.02 × 10^−11^
0.1Li-DC-1.0A	85.98%	1498	1338	1.61 × 10^−9^
0.1Li-AC-0.8A	98.64%	1700	1394	8.41 × 10^−9^
0.3Li Green	64.83%			
0.3Li-DC-0.8A	72.50%			4.65 × 10^−9^
0.3Li-DC-1.0A	80.43%	1284	1023	3.77 × 10^−9^
0.3Li-AC-0.8A	94.66%	1684	1256	2.02 × 10^−9^

## Data Availability

Not applicable.
